# Evaluating the Sustainable Land Use in Ecologically Fragile Regions: A Case Study of the Yellow River Basin in China

**DOI:** 10.3390/ijerph19063222

**Published:** 2022-03-09

**Authors:** Wentao Niu, Jingyi Shi, Zhenzhen Xu, Tianxi Wang, Hexiong Zhang, Xiaoshan Su

**Affiliations:** 1 Research Center for Economic Development and Environment of the Yellow River Basin, Zhengzhou University, Zhengzhou 450001, China; wentaoniu@zzu.edu.cn (W.N.); shi__jingyi@163.com (J.S.); 2 School of Tourism Management, Zhengzhou University, Zhengzhou 450001, China; 3 School of Architecture and Built Environment, Deakin University, Geelong 3219, Australia; xuzhenz@deakin.edu.au; 4 Business School, University of Edinburgh, 29 Buccleuch Place, Edinburgh EH8 9JS, UK; s1819714@ed.ac.uk; 5 College of Public Administration, Central China Normal University, Wuhan 430079, China; 15333868696@163.com

**Keywords:** Yellow River Basin, central cities, sustainable land use, obstacle factors, China

## Abstract

How to realize the sustainable use of land resources is extremely important for environmental protection and sustainable development in ecologically fragile regions. Nevertheless, the logic of achieving sustainable land use (SLU) in ecologically fragile regions and the corrective mechanisms for the implementation of land use efficiency systems are not fully revealed in theory. The Yellow River Basin is an important ecological barrier in China, and it holds an important position in China’s economic and social development, as well as for ecological safety. However, the basin is also ecologically vulnerable. Therefore, investigating eight central cities in the Yellow River Basin of China and using municipal-level panel data from 2009 to 2018, this paper constructs a multidimensional index system and is dedicated to carrying out a comprehensive evaluation of SLU and the diagnosis of obstacle factors in ecologically fragile regions. The study found the following: (1) From 2009 to 2018, the SLU level in the central cities of the Yellow River Basin evolved from the “Unsustainable Level” to the “Initial Sustainable Level” and then to the “Basic Sustainable Level”. The overall development trend was positive, and the level of SLU also rose. (2) From 2009 to 2018, there was significant geographical variation in spatial disparities in SLU in the central cities of the Yellow River Basin. In 2018, the average comprehensive score of SLU showed a pattern of downstream > upstream > midstream. (3) The obstacle factors of SLU in the Yellow River Basin of these cities in 2009 were concentrated on resource and environmental sustainability, while those in 2018 were concentrated on social acceptability. (4) In terms of the transfer process of land use types in these Yellow River Basin cities, the transfer from cultivated land to other types of land use played a major role, while construction land showed a significant expansion over the past ten years.

## 1. Introduction

Land resources are the material basis for the survival and development of human society and the source of the creation of other social wealth. The sustainable use of land is a prerequisite and foundation for the sustainable development of social economy and resources and environment [1]. With the rise and prevalence of sustainable development awareness, sustainable land use (SLU) has become an important element in the sustainable development of human society. The evaluation of SLU originated from the evaluation of land potential, land classification and grading, and evaluation of land suitability in the 20th century [2], and with the deepening of the concept of sustainable development, the academic community began to extend the idea of sustainability to land use and other fields in the 1990s. The idea of SLU was first introduced at the International Symposium on Sustainable Land Use Systems in 1990 [3]. The International Symposium on Sustainable Land Use Evaluation in Developing Countries in 1991 and the International Symposium on Sustainable Land Management in the 21st Century in 1992 boosted more in-depth research and discussions on sustainable land use and initiated indexes for the evaluation of sustainable land use from natural, economic and social aspects. The importance of constructing an evaluation index system for SLU was confirmed. In addition, in 1993 the Food and Agriculture Organization of the United Nations (FAO) defined SLU as a land use that is considered sustainable if it is predicted not to cause the degradation of land suitability for a significant period of time in the future. At the same time, the FAO published the Outline of Sustainable Land Evaluation in 1993, which specified the procedures, basic principles and evaluation criteria for SLU [4]. At this stage, the proper use and development of limited land resources and the improvement and protection of land resources raised widespread concern [5]. On this basis, sustainable land resource use assessment is an important tool to ensure that land use is on a sustainable development trajectory [6]. Quantitative analysis for SLU assessment helps to understand the current situation of land use, to analyze relevant issues in a more intuitive way and to make targeted recommendations for rational future development. Therefore, research into the evaluation of SLU is both relevant and urgent.

The evaluation of SLU has been studied since the late 20th century. For example, John et al. study the SLU in Machakos, Kenya [7]; Gameda et al. evaluate the SLU in Canada at the farm level [8]; in addition, by analyzing the characteristics of SLU, some scholars establish an index system to evaluate SLU in terms of three aspects: ecological, economic and social [9]. At the beginning of the 21st century, the development of a land resource sustainability index system was a priority study area in science and technology proposed to countries in Agenda 21. Some scholars explore the theory and methodology of a sustainable land resource use index system in China as a whole [10] and continue to develop new analytical frameworks for land sustainability theory [11,12]. Some scholars use sustainable development theory [5] and the United Nations 2030 Sustainable Development Goals (SDGs) [13] as the basis for an index system to evaluate the development status of SLU [6,14]. However, as existing assessment methods and corresponding indexes may not provide a comprehensive assessment of sustainability practices, some qualitative and integrated approaches are needed to complement SLU assessment. These approaches contain, for example, the development of integrated assessment frameworks that include expert and public input [15], the inclusion of relevant natural and economic indexes with a view to an integrated evaluation of SLU [16] and the inclusion of landscape ecological indexes that contribute to the spatial evaluation of SLU [17,18]. In addition, the goals of land sustainability, such as gender equality, cross-region coordination and land property protection, are receiving increasing attention worldwide [11], and the assessment of land use suitability for sustainable construction can contribute to higher sustainable development levels [19,20].

The existing research on SLU evaluation can be divided into three major dimensions. First, scholars study the evaluation of SLU in provinces [21,22,23], cities [24,25,26,27,28,29,30] or regions [12,13,31,32,33,34,35,36,37]. They construct a set of index systems for SLU evaluation to cater for regional variation and to conduct quantitative analysis. Secondly, in recent years, scholars have innovatively extended their research to the spatial and temporal analysis of SLU [13,23,27,29,30,33,36,37], cluster analysis [38], obstacle analysis [39], hot spot analysis [40] and coordination analysis [41], etc. They analyze and evaluate SLU from multiple perspectives in different studies, thus proposing countermeasures suitable for different study areas. Third, scholars develop their methodologies mainly based on the entropy method [39,42,43], a principal component analysis method [37,40,44], a PSR model [41,45,46,47,48,49], an improved TOPSIS method [50], a hierarchical analysis method [21,27,38], etc. Most studies develop new research methodologies for SLU evaluation or improve previous studies. These studies provide policy implications on SLU in the study area.

In general, the current research on sustainable urban land use is mainly conducted at the province or city level. Some studies take the Yellow River Basin as the study area [51,52,53,54]. However, studies using central cities of the Yellow River Basin as the observation dimension are relatively scarce. The Yellow River Basin is an important ecological barrier in China, and it holds an important position in China’s economic and social development and ecological safety. However, this area is ecologically vulnerable. Over the past seventy-two years, since the founding of the People’s Republic of China, great achievements have been made in the governance of the Yellow River, and the central cities in the Yellow River Basin. These cities, represented by Zhengzhou, Xi’an and Jinan, have gained remarkable development in terms of urban landscape and people’s living standards. In this process, these cities’ urban land use efficiency system shows a significant “socio-economic efficiency preference”, which puts a negative constraint on the overall ecological environment and the high-quality economic and social development of the Yellow River Basin in the long term. Therefore, it is urgent to actively implement ecological environmental protection and high-quality development strategies in the Yellow River Basin, to explore the logic of SLU in the central cities in this area, to develop a mechanism for correcting the systemic bias of urban land use in ecologically fragile regions, and to clarify the inner working mechanism of ecological environmental protection and high-quality economic and social development in the Yellow River Basin.

This paper investigates eight central cities in the Yellow River Basin and uses the entropy method, weighted method and obstacle factor analysis method. In this study, eighteen indexes in three categories, namely, economic feasibility, social acceptability and resource and environmental sustainability, are selected to develop an evaluation index system for SLU. In addition, this paper combines the data on the land use of eight central cities in the Yellow River Basin from 2009 to 2018 to carry out a comprehensive evaluation of SLU and a diagnostic study of the obstacle factors. It reveals the logic of achieving SLU in the central cities of the Yellow River Basin and constructs a mechanism for correcting the operational bias of the urban land use efficiency system in ecologically fragile regions.

## 2. Study Area and Data Sources

### 2.1. Study Area

**Figure 1:**
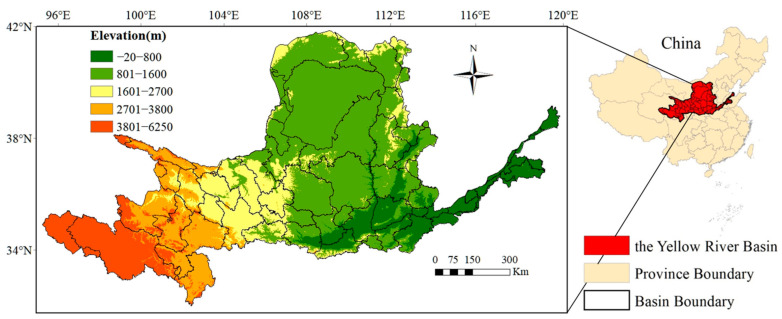
Topographic location of the Yellow River Basin, China.

The Yellow River Basin (as shown in Figure 1), from west to east, spans four geomorphic units: the Tibetan Plateau, the Inner Mongolia Plateau, the Loess Plateau and the Yellow Huaihai Plain. The Yellow River originates in the Bayankara Mountains in Qinghai Province of China; flows through nine province-level regions, namely Qinghai, Sichuan, Gansu, Ningxia, Inner Mongolia, Shaanxi, Shanxi, Henan and Shandong; and finally flows into Bohai in Dongying, Shandong Province. The Yellow River has a total length of 5464 km and a basin area of 752,443 km^2^. The Yellow River Basin is divided into upper, middle and lower reaches, with the dividing points being Hekou Town in Inner Mongolia and Taohuayu in Henan Province. The Yellow River Basin is in the mid-latitude zone and covers the southern temperate zone, the middle temperate zone and the plateau climate zone. It has sufficient sunlight, a large variation in seasons and temperature variation. The seasonal precipitation is concentrated in June to September and accounts for approximately 70% of the whole-year precipitation.

The Yellow River Basin is an agriculturally and economically active area in China. With the Loop Plain in the upper reaches, the Fenwei Basin in the middle reaches and the Yellow Irrigation Area in the lower reaches, this basin is one of the major agricultural production bases. The upper and middle reaches of the Yellow River Basin are still relatively underdeveloped, but the basin is rich in natural resources, such as hydro energy resources in the upper reaches, coal resources in the middle reaches, and oil and gas resources in the lower reaches. Since 1960, several water conservancy hubs, such as Sanmenxia, Sanshenggong, Qingtongxia and Liujiaxia, have been put into use one by one. In 2019, Chinese President Xi Jinping convened a symposium on ecological protection and the high-quality development of the Yellow River Basin, emphasizing their importance. Among the cities of this area, rapid economic development and urban expansion have some negative impacts on land resource use and the ecological environment, although people have also paid increasing attention to ecological protection. Therefore, the evaluation of SLU will be helpful to outline the current situation and to solve the problems of SLU in the central cities of the basin in a more comprehensive way and put forward targeted policy recommendations, which are of great significance to their sustainable development. Given the data availability and the representativeness of sample selection, eight cities, namely, Xining, Lanzhou, Yinchuan, Hohhot, Taiyuan, Xi’an, Zhengzhou and Jinan, were selected as the research object of this paper.

### 2.2. Data Sources

The data used in this study mainly consist of the following three parts: First, the dataset of the evaluation index consists of annual data from 2009 to 2018. It contains eighteen indexes on social acceptability, economic feasibility and resource and environmental sustainability of eight central cities in the Yellow River Basin. Among them, the data of the natural population growth rate, population density, grain production, disposable income per capita, GDP per capita, investment in fixed assets, total retail sales of consumer goods, public finance budget revenue, share of tertiary sector in GDP, real estate development investment, green coverage rate of built-up areas, sewage treatment rate, industrial solid waste generation, park green area per capita, fertilizer application amount and other related indexes are mainly drawn from the Statistical Yearbooks of the tight central cities [55,56,57,58,59,60,61,62]. The index of water resources is from the Water Resources Bulletin of these cities for each year. The urbanization rates of the resident population in Xining, Yinchuan, Hohhot, Taiyuan and Xi’an, and the Engel coefficient in Lanzhou and Jinan, were obtained from the Statistical Bulletin of National Economic and Social Development, while the data of other cities were obtained from their corresponding Statistical Yearbooks. Second, the ArcGIS remote sensing images and the vector boundary data of eight cities were obtained from https://www.resdc.cn, (accessed on 3 March 2022) [63]. Given the unavailability of the vector boundary data in 2009, the time frame for land use transition matrix analysis in this study was 2010–2020. Third, the Chinese altitude (DEM) spatial distribution data, generated by resampling of US SRTM (Shuttle Radar Topography Mission) data from the Resource and Environment Science and Data Center (https://www.resdc.cn (accessed on 3 March 2022)) [63]. The data were resampled to a resolution of 250 m × 250 m.

## 3. Research Methodology

### 3.1. Evaluation Index System Construction

According to the principles of the scientific method, feasibility, and dynamics of evaluation index selection, an evaluation index system was constructed (as shown in Table 1). The system has three levels, which are the target layer, rule layer and index layer. The target layer is SLU. The rule layer was further divided into three sublayers: social acceptability, economic feasibility and resource and environmental sustainability; each of the three sublayers correspond to six indexes in the eighteen indexes of the index layer.

#### 3.1.1. Social Acceptability

SLU must match the current situation of society while conforming to the basic laws of social development. In this paper, six indexes were selected in terms of social acceptability to evaluate the SLU in the central cities of the Yellow River Basin. The six indexes were natural population growth rate, population density, urbanization rate of resident population, grain production, disposable income per capita and Engel coefficient. The natural population growth rate, population density and Engel coefficient are inverse indexes, while the urbanization rate of resident population, grain production and disposable income per capita were positive indexes.

#### 3.1.2. Economic Feasibility

There is a typical two-way interacting relation between the level of economic development and the level of SLU. In this paper, six indexes were selected in terms of economic feasibility to evaluate the SLU in the central cities of the Yellow River Basin. The six indexes were GDP per capita, investment in fixed assets, total retail sales of consumer goods, public finance budget revenue, share of tertiary sector in GDP and real estate development investment. These six indexes were all positive indexes.

#### 3.1.3. Resource and Environmental Sustainability

Social acceptability and economic feasibility are social attributes in the process of SLU, while resource and environmental sustainability are natural attributes in the process of SLU. In this paper, six indexes were obtained to evaluate the SLU in the selected cities in terms of resource and environmental sustainability. The six indexes were green coverage rate of built-up areas, sewage treatment rate, industrial solid waste generation, park green area per capita, fertilizer application amount, and water resources per capita. Among them, industrial solid waste generation and fertilizer application amount were inverse indexes; the green coverage rate of built-up areas, sewage treatment rate, park green area per capita and water resources per capita were positive indexes.

### 3.2. Quantitative Evaluation of SLU

#### 3.2.1. Entropy Method to Calculate the Weight of Each Index

The entropy method can measure the degree of dispersion of a certain index. The greater the degree of dispersion, the greater the influence of the index on the comprehensive evaluation. Therefore, the information contained in the entropy value can be used for weight calculation, and the tool of information entropy can be used to calculate the weights of each index in combination with the degree of variation of each index. The outputs of the calculation provide a basis for the comprehensive evaluation of multiple indexes.

The first step was data standardization. Since the units of each index were different, standardizing the data before calculating the composite indexes can mitigate the homogenization among different qualitative indexes and make the indexes more comparable. Since the values of positive and negative indexes have different meanings, they were calculated using different formulas. Positive and negative indexes were calculated with Equations (1) and (2) below:

(1) $$A_{ij}=\frac{X_{ij}-X_{j\min}}{X_{j\max}-X_{j\min}}$$

(2) $$A_{ij}=\frac{X_{j\max}-X_{ij}}{X_{j\max}-X_{j\min}}$$

where $X_{ij}$ denotes the value of the jth index in the ith year; $A_{ij}$ denotes the value of the jth index in the ith year after standardization.

The second step was to calculate the entropy value of the jth index. Before calculating the entropy values, the normalized values were shifted by 0.01 units in order to avoid the situation where ln0 is meaningless, without affecting the results of the operation. Subsequently, the entropy value of the jth index was calculated using the following Equation (3):

(3) $$\begin{array}{l} S_{j}=\frac{-\sum_{i=1}^{n}B_{ij}\ln B_{ij}}{\ln_{n}}\\ B_{ij}=\frac{A_{ij}}{\sum A_{ij}} \end{array}$$

where $S_{j}$ denotes the entropy value; $B_{ij}$ denotes the weight of the value of the jth index in the ith year; $A_{ij}$ denotes the value of the jth index in the ith year after standardization; n denotes the number of years; m denotes the number of evaluation indexes.

The third step was the calculation of the index weights. The calculation method is as follows in Equation (4):

(4) $$W_{j}=\frac{\left(1-S_{j}\right)}{\sum \left(1-S_{j}\right)}$$

where $W_{j}$ denotes the weight of the index layer where the jth index is located; $1-S_{j}$ denotes the coefficient of variation.

The coefficient of variation measures the differences between indexes. A smaller entropy value means a larger coefficient of variation, as well as a greater role of indexes, and vice versa.

The entropy value of each index was derived from Equation (3), and the weight of each index was derived from Equation (4) (as shown in Table 2). The weight of the rule layer could be calculated from the sum of the weight of each rule layer corresponding to the weight of the index layer.

#### 3.2.2. Comprehensive Evaluation of SLU

After calculating the weight of each index according to Equations (1)–(4), the weighted method was applied to calculate the comprehensive score value. The calculation method is as follows in Equation (5):

(5) $$F=\sum_{i=1}^{n}W_{j}A_{ij}$$

where F denotes the comprehensive score value; $W_{j}$ denotes the weight of the index layer where the jth index is located; $A_{ij}$ denotes the value of the jth index in the ith year after standardization; n denotes the number of years.

The comprehensive score value of SLU was then classified into four grades with reference to the Evaluation Index System and Method of SLU [64] to obtain the evaluation criteria for the SLU level in this paper (as shown in Table 3). In this way, the specific levels of SLU in eight central cities in the Yellow River Basin could be measured.

#### 3.2.3. Obstacle Degree to SLU

In the process of sustainable urban land use, some factors may hinder the sustainable use of land and constitute so-called “obstacle factors” for sustainable urban land use. These factors eventually produce certain negative constraints on the realization of the comprehensive benefits of urban land use. In order to effectively identify these obstacle factors and to develop a mechanism to solve the urban land use dilemma, it is necessary to diagnose and measure the obstacle degree of these factors. First, the factor contribution rate was calculated using Equation (6):

(6) $$T_{j}=V_{j}\times W_{j}$$

where $T_{j}$ denotes the factor contribution rate; $V_{j}$ denotes the weight of the rule layer where the jth index is located; $W_{j}$ denotes the weight of the index layer where the jth index is located.

Second, the deviation degree of the index was calculated using Equation (7):

(7) $$L_{ij}=1-A_{ij}$$

where $L_{ij}$ denotes the deviation degree of the index; $A_{ij}$ denotes the value of the jth index in the ith year after standardization.

Third, the obstacle degree of sustainable use was calculated using Equation (8):

(8) $$Z_{ij}=\frac{T_{j}L_{ij}}{\sum_{j-1}^{m}T_{j}L_{ij}}$$

where $Z_{ij}$ denotes the obstacle degree of sustainable use; $T_{j}$ denotes the factor contribution rate; $L_{ij}$ denotes the deviation degree of the index; m denotes the number of evaluation indexes.

#### 3.2.4. Land Use Transfer Matrix

The sustainable use of urban land is closely related to the change in land use type, and whether the change in land use type is appropriate or not will directly affect the level of SLU. The land use transfer matrix can quantify the transfer between different land use types and show the structural characteristics of land use change more comprehensively, which is conducive to the in-depth analysis of various land use type changes. In this paper, the confusion matrix of the ENVI5.3 platform was used to calculate the land use transfer matrix, so as to reflect the land use change information of eight central cities in the Yellow River Basin from the 10 years from 2010 to 2020, and to provide a reference for how to influence and improve the SLU level after the land use type change.

## 4. Results

### 4.1. Time Series Analysis

Through the work of time series analysis of SLU in the central cities of the Yellow River Basin, the evolution process of SLU in these cities at the time scale and the basic laws were revealed. Based on Equation (5), a comprehensive score for SLU in the central cities of the Yellow River Basin can be obtained (as shown in Table 4). The score can be used to determine the level of SLU in each city. The comprehensive score varies between 0 and 1. A higher score indicates a higher level of SLU and vice versa. With the identical method, the score of each rule layer for SLU in these cities (as shown in Table 5) can be obtained. It is equal to the sum of the scores of each index layer. The sum of the scores of each rule layer is the comprehensive score. The score of each rule layer measures the weights of social, economic, resource and environment aspects in each city’s land sustainability level.

Based on the evaluation criteria for the SLU level (as shown in Table 3) and the comprehensive score of the SLU indexes for central cities in the Yellow River Basin (as shown in Table 4), we discuss the evolution of SLU in these cities over the years. The level of SLU in the central cities of the Yellow River Basin was continuously optimized during the period 2009–2014. All eight central cities were at “Unsustainable Levels” in 2009 and remained so until the end of 2012. This indicates that the central cities in the Yellow River Basin demonstrated careless and inefficient land use during the period 2009–2012, which is consistent with the findings of Li et al. (2019) [39]. The improvement of China’s international status, China’s membership in WTO, and the reform of the market economy induced rapid economic development, but also led to intensified environmental pollution and the inappropriate use of resources and energy. These caused serious ecological problems and damaged resources and the environment. Therefore, during the period 2009–2012, China witnessed both rapid economic growth and the destruction of resources and environments, which hindered the improvement of SLU in the Yellow River Basin. Although the level of SLU in the central cities of the Yellow River Basin was at the “Unsustainable Level” during this period, the comprehensive score of SLU in each city showed a steady increase. The comprehensive score of each city in 2012 increased substantially by an average of 40.1% compared to the comprehensive score in 2009. In 2013, the SLU in the eight central cities of the Yellow River Basin improved significantly. Hohhot was the first city to reach the “Initial Sustainability Level” and remained the only city at this level until 2014. The main reason for this was the rapid economic development of Hohhot under the strong guidance and cooperation of the central government and local government. In 2013, the city’s economic feasibility score reached 0.275, which was highest among the eight central cities in that year. Hohhot also had the highest public finance budget revenue and real estate development investment in the period 2009–2018, making it the first city among the eight central cities to reach the “Initial Sustainability Level”.

The level of SLU in the central cities of the Yellow River Basin showed a continuous improvement from 2015 to 2017. In 2015, in addition to Hohhot, three other cities, Xining, Taiyuan and Xi’an, also reached the “Initial Sustainability Level”, which is consistent with the findings of Jiao et al. (2019) [37] In 2016, all of the eight central cities reached the “Initial Sustainability Level”, which is a milestone in SLU. As the eastern gateway to the Qinghai-Tibet Plateau, the only central city with a population of more than one million on the plateau, and the essential route of the ancient Silk Road and the Tangfan Ancient Road, Xining made full use of its endowments in terms of geographical location and natural resources. With external policy supports, it maintained a high growth rate for all indexes in general. In 2016, Xining became the first city among the eight central cities to reach the “Basic Sustainability Level”. The city’s resource and environmental sustainability score in that year reached 0.283, which was the highest among the eight central cities at that time. This is mainly attributed to the fact that industrial solid waste generation and fertilizer application were effectively controlled, and the fertilizer application amount decreased by 27.4% compared to the previous year. In 2017, in addition to Xining, four other cities, Lanzhou, Taiyuan, Zhengzhou and Jinan, also reached the “Basic Sustainability Level”. Among them, except Hohhot, of which the comprehensive score of SLU fell in 2017, the comprehensive scores of SLU in other cities showed a common upward trend. This was mainly due to the low economic feasibility score of Hohhot in 2017 (0.188), which decreased by 32.9% compared to the previous year. Among them, two indexes, investment in fixed asset and real estate development investment, fell by 19.4% and 54.2%, respectively, leading to a decrease in the comprehensive score of SLU in Hohhot in 2017 compared to 2016.

The SLU levels of the eight central cities in the Yellow River Basin were all at a high level in 2018, while Jinan was the only city at the “Fully Sustainable Level” in 2018. In 2016, the State Council listed Jinan as the third batch of national comprehensive pilot areas for new urbanization, and the SLU level in Jinan was at the “Basic Sustainable Level” in 2016. The comprehensive score of SLU in Jinan increased year by year, and in 2018, the score of social acceptability in Jinan reached 0.323, which was the highest among the eight central cities in that year. Among them, the urbanization rate of the resident population reached 84.48%, with an increase of 12.4% over the previous year. The disposable income per capita of Jinan also continued to rise, exceeding CNY 50,000, which was the highest among the eight central cities in that year. As a result, Jinan became the first city in the Yellow River Basin to reach the “Fully Sustainable Level” in 2018. In 2018, there were six cities at the “Basic Sustainable Level”, namely Xining, Lanzhou, Yinchuan, Taiyuan, Xi’an and Zhengzhou. Among them, Xining, Lanzhou and Zhengzhou had a comprehensive score of more than 0.8, with high scores for economic feasibility and resource and environmental sustainability and relatively low scores in social acceptability. The main reason for this was the implementation of the 13th Five-Year Plan and the government’s proposal to reform the economic development mode, leading to a rapid increase in the level of high-quality economic development. Meanwhile, the government advocated resource and technology intensive industries and focused on the construction of ecological civilization, which greatly improved the sustainability of resources and environments. High-quality economic development and enhanced resource and environmental sustainability provided a good impetus for the improvement of SLU in the central cities of the Yellow River Basin. Only Hohhot remained at the “Initial Sustainable Level” in 2018. The comprehensive score of SLU in Hohhot in 2016 was 0.690, which was among the highest during the period 2009–2018. Since then, Hohhot’s economy has declined, with two indexes, investment in fixed assets and real estate development investment, continuing to fall. Investment in fixed assets and real estate development investment decreased by 26.5% and 26.9%, respectively in 2018 compared to the previous year. Moreover, Hohhot’s GDP per capita also decreased by 15.9% in 2018 compared to 2017, which showed that the economic downturn seriously hindered the improvement of SLU levels in Hohhot.

### 4.2. Spatial Disparity Analysis

**Figure 2:**
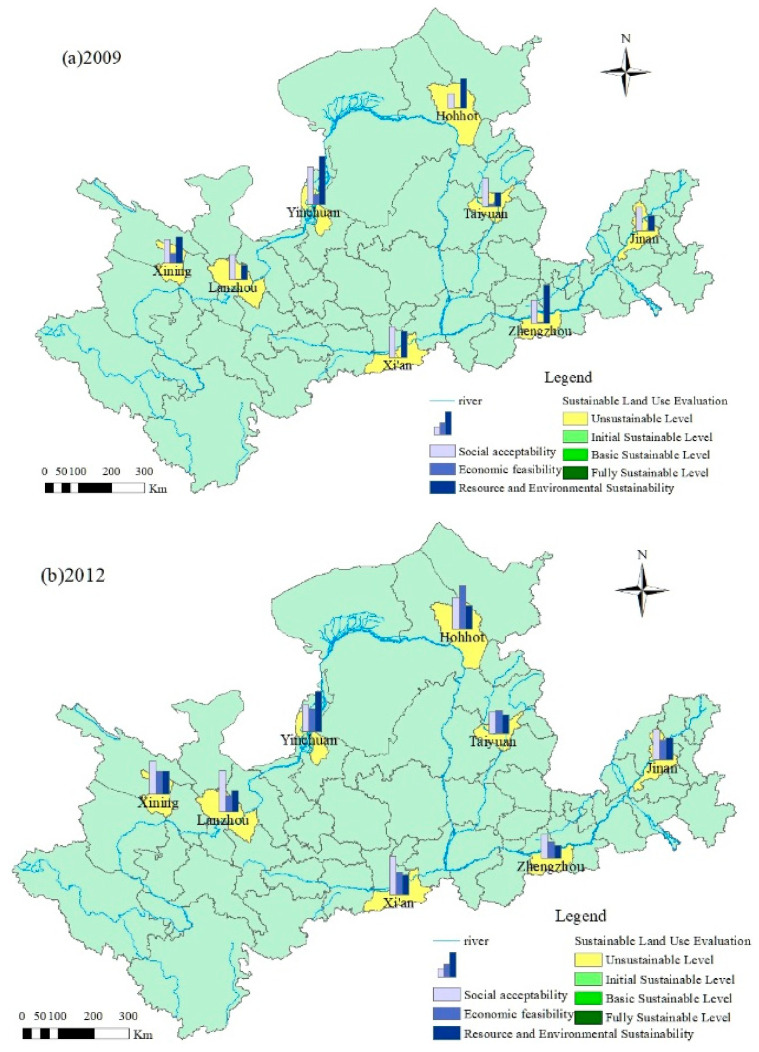

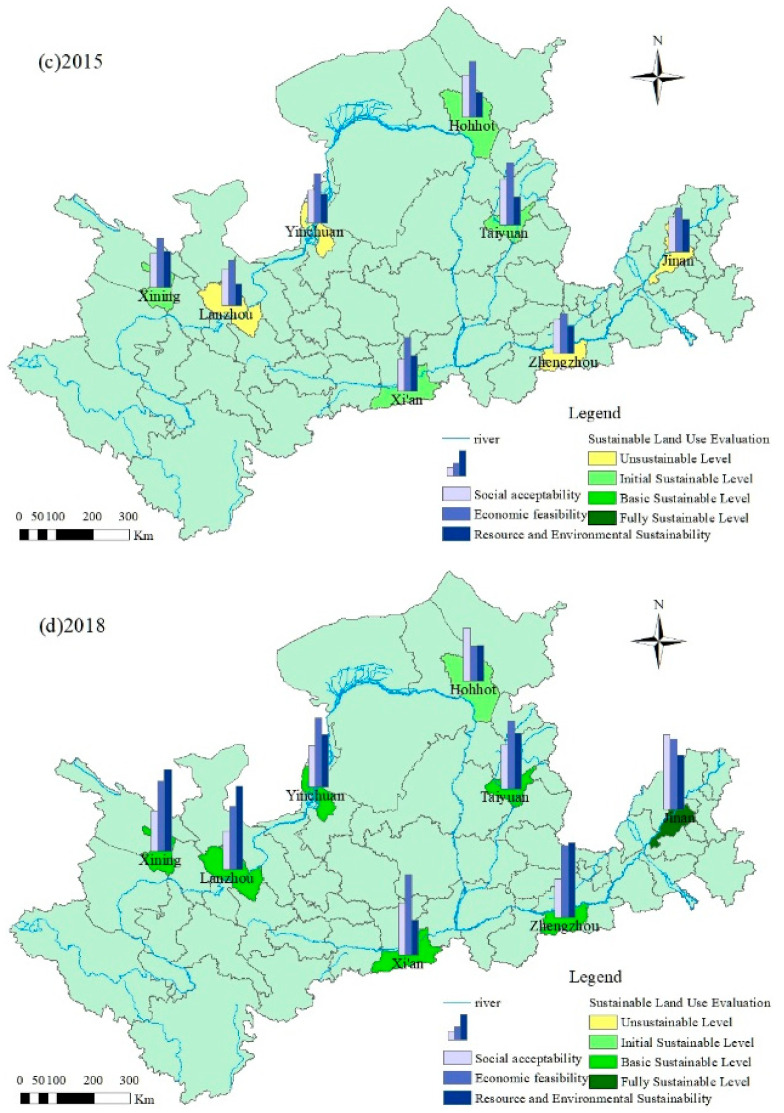
Analysis of spatial disparities in SLU in central cities of the Yellow River Basin, China.

According to the data in the evaluation criteria for the SLU level (as shown in Table 3), the comprehensive score of SLU indexes for central cities in the Yellow River Basin (as shown in Table 4) and the score of each rule level for SLU in these cities (as shown in Table 5), four years (2009, 2012, 2015, and 2018) were selected, and ArcGIS spatial analysis techniques were used to carry out the analysis of spatial disparities in SLU (as shown in Figure 2). This revealed the spatial evolution of SLU levels in the eight cities.

In terms of the overall development trend, the SLU levels of the eight central cities in the Yellow River Basin evolved from the “Unsustainable Level” to the “Basic Sustainable Level” from 2009 to 2018, which is consistent with the findings of Wang et al. (2018) [41] Among them, only Jinan reached the “Fully Sustainable Level”. In 2009, the SLU levels of the eight central cities in the Yellow River Basin were all at the “Unsustainable Level”, and the problem of inappropriate inefficient land use was highlighted. Although the SLU level increased in 2012, it was still in the “Unsustainable Level”. In 2015, Xining, Hohhot, Taiyuan and Xi’an reached the “Initial Sustainable Level” and the level of SLU had improved significantly; in 2018, only Jinan reached the “Fully Sustainable Level”, but Hohhot still remained at the “Initial Sustainable Level”, and the remaining six cities were at the “Basic Sustainable Level”.

In terms of the spatial evolution trend, from 2009 to 2018, there was significant geographical variation in spatial disparities in SLU in the central cities of the Yellow River Basin, which is consistent with the findings of Jiao et al. (2019) [37] and Wang et al. (2018) [41] Before 2015, only Hohhot, located in the middle reaches, among all of the eight central cities in the upper, middle and lower reaches of the Yellow River Basin, took the lead in reaching the “Initial Sustainable Level”, while the remaining seven cities were all at the “Unsustainable Level”, and the middle reaches were the first to have a breakthrough at the SLU level. In 2015, the average comprehensive scores of SLU were 0.485 for three cities in the upper reaches, 0.548 for three cities in the middle reaches and 0.463 for two cities in the lower reaches; the average comprehensive score of SLU showed a pattern of midstream > upstream > downstream. Since then, due to the continuous economic development in the downstream regions and the emphasis on ecological improvement, the resources and environment were significantly optimized, and the scores of economic feasibility and resource and environmental sustainability of the two downstream cities also significantly increased. As of 2018, the average value of the comprehensive score of SLU in the downstream region reached 0.834, ranking first; the average values of the comprehensive score of SLU in the upstream region and the midstream region were 0.783 and 0.662, respectively, and the average value of the comprehensive score of SLU showed a pattern of downstream > upstream > midstream. The midstream region’s relatively poor ecological background, the lack of awareness of resources and environment, and the decline in the comprehensive score of Hohhot limited the improvement of the SLU level in the midstream region, making it fall from first to third in 2015. This showed that as of 2018, the sustainable use of upstream and downstream central cities had improved significantly faster than that of midstream central cities.

The rule layer characteristics also warrant further discussion. From 2009 to 2012, although the SLU level of the central cities in the Yellow River Basin was at the “Unsustainable Level”, their SLU scores at the rule layer showed an overall increase, which is consistent with the findings of Su et al. (2018) [44] The social acceptability score showed a slow general increase. The average score in 2018 was approximately 1.7 times that of 2009. The main reason for this was that the two indexes of population density and natural population growth rate continued to rise, restricting the increase in the social acceptability score. Meanwhile, the economic feasibility score was generally on the rise, which is consistent with the findings of Han et al. (2018) [50] After 2012, the economic feasibility score increased significantly, and the average score in 2018 was approximately 18.4 times higher than that in 2009. In 2015, the economic feasibility score was the major driver of the comprehensive score of SLU in the eight cities. Moreover, although the resource and environmental sustainability score showed an upward trend, it grew slowly. The average score in 2018 was approximately twice that of 2009. In 2018, the score of resource and environmental sustainability in Xining, Lanzhou and Zhengzhou was higher than that of economic feasibility.

### 4.3. Diagnostic Analysis of Obstacle Factors

Based on Equations (6)–(8), the obstacle degree of SLU indexes (as shown in Table 6) was measured to identify the main constraints on SLU in the eight central cities in the Yellow River Basin. As shown in Table 6, the factors that hindered the improvement of SLU in the cities in 2009 were mainly focused on the resource and environmental sustainability, mainly including the sewage treatment rate, park green area per capita, and the green coverage rate of built-up areas. Among them, the index of the sewage treatment rate hindered the improvement of SLU in five cities in the Yellow River Basin, namely Xining, Yinchuan, Hohhot, Taiyuan and Jinan. In 2009, the average obstacle degree was as high as 30.7% among the five cities.

The SLU is closely related to resource and environmental sustainability. The sewage treatment rate, park green area per capita and green coverage rate of built-up areas directly affect the level of sustainable urban land use. In 2009, the sewage treatment rate of Zhengzhou and Xi’an reached 97.2% and 80.97%, respectively, while the figures for Xining and Hohhot were only 54.47% and 57%, respectively. The low sewage treatment rate seriously limited the improvement of the SLU level. In 2009, the park green area per capita in Xi’an was only 7.9 m^2^, the green coverage rate of built-up areas in Zhengzhou was only 34.5% and the two indexes increasingly rose after 2009. As a result, the park green area per capita in Xi’an and the green coverage rate of built-up areas in Zhengzhou had the highest obstacle degree in 2009. The factors that hindered the improvement of SLU in the central cities of the Yellow River Basin in 2018 were mainly focused on social acceptability, including population density, grain production and natural population growth rate. Among them, the index of population density in 2018 hindered the improvement of SLU in three cities in the Yellow River Basin, namely Xining, Hohhot, and Xi’an, with a high average obstacle degree of 27.19%. The findings of this paper regarding the obstacle degree are inconsistent with those of Li et al. (2019) [39] Li et al. (2019) identified the obstacle factors to SLU in the suburbs of Jinan in the following order: cultivated land area per capita, fixed assets per capita, waste harmless treatment rate and sewage treatment rate. The different conclusions of the study may have been caused by the different study areas and study years. The study by Li et al. (2019) focused on the period 2009–2017, and the study area was only Jinan City; this paper is a study of eight central cities in the Yellow River basin from 2009 to 2018.

SLU is also closely related to social acceptability. Population density, grain production, and natural population growth rate influence the level of sustainable urban land use. Among the central cities in the Yellow River Basin, except for Hohhot, the grain production decreased in all of the remaining seven cities. In particular, the grain production of Lanzhou and Taiyuan in 2018 was only 297,700 t and 292,300 t, respectively, while the grain production of Jinan in the same year was 2,514,200 t. The population density and natural population growth rate also put great pressure on SLU. Xining, Hohhot, and Xi’an had the largest population density obstacle degree, while the natural population growth rate in Zhengzhou continued to grow from 2009 to 2018 and reached 7‰ in 2018, making it the main restricting factor for SLU in Zhengzhou in 2018. It follows that improving SLU in the central cities of the Yellow River Basin must start with resource and environmental sustainability and social acceptability, while focusing on improving economic feasibility, a finding consistent with that of Han et al. (2018) [50]

### 4.4. Land Use Transfer Matrix Analysis

By interpreting remote sensing images using the ENVI platform, the land use types of central cities in the Yellow River basin were mainly classified into five categories: cultivated land, forest land, grassland, water area and construction land. In addition to the five major categories, Xining has two additional categories of land use, bare land and glacial snow; Lanzhou, Yinchuan, Hohhot and Jinan have the additional category of bare land. Through a further analysis of the land use transfer matrix, the change in land use types in the central cities of the Yellow River Basin during the period 2010 to 2020 could be evaluated more clearly. Table 7, Table 8, Table 9, Table 10, Table 11, Table 12, Table 13 and Table 14 below show the land use transfer matrix for the central cities in the Yellow River Basin during the ten-year period.

Based on the land use transfer matrix in Xining during this period (Table 7), it was found that the major land use type conversions in Xining occurred in forest land and glacial snow. Among them, 32.60% of the forest land was converted to grassland, totaling 2.74 km^2^; 24.86% of the glacial snow was converted to grassland, totaling 17.18 km^2^. The construction land area of Xining increased from 168.48 km^2^ to 457.73 km^2^ over the decade, with a growth rate of 171.67%. The increase in construction land was mainly due to occupying cultivated land, and 257.10 km^2^ of cultivated land was converted to construction land over the decade.

As can be seen from Table 8, the major land use type conversions in Lanzhou during the period 2010 to 2020 took place in forest land and bare land. Among them, 67.96% of the forest land was converted to grassland, totaling 156.76 km^2^; 42.19% of the bare land was converted to grassland, totaling 29.69 km^2^. The construction land area of Lanzhou increased from 199.80 km^2^ to 769.87 km^2^ over the decade, with a growth rate of 285.32%. The increase in construction land was mainly due to occupying cultivated land, and 450.69 km^2^ of cultivated land was converted to construction land over the decade.

As shown in Table 9, the largest land use type conversions in Yinchuan during the period 2010 to 2020 were forest land and water area. Among them, 28.79% of the forest land was converted to grassland, totaling 54.72 km^2^; 31.79% of the water area was converted to cultivated land, totaling 36.89 km^2^. The construction land area of Yinchuan increased from 233.72 km^2^ to 667.31 km^2^ over the decade, with a growth rate of 185.52%. The increase in construction land was mainly due to occupying cultivated land and grassland, with 295.74 km^2^ of cultivated land converted to construction land and 137.32 km^2^ of grassland converted to construction land over the decade.

Compared with the cities analyzed above, Hohhot’s pattern of land use type conversion over the decade was distinct to that of others. Pursuant to Table 10, the most significant land use type conversion in Hohhot during the period 2010 to 2020 was bare land; 50.75% of the bare land was converted to grassland, totaling 15.64 km^2^. The construction land area of Hohhot increased from 722.30 km^2^ to 1039.82 km^2^ over the decade, with a growth rate of 43.96%. The increase in construction land was mainly due to occupying cultivated land and grassland, with 352.12 km^2^ of cultivated land converted to construction land and 105.10 km^2^ of grassland converted to construction land in those ten years. The city also converted 129.57 km^2^ of construction land to cultivated land. Moreover, the cultivated land area and grassland area had basically reached a requisition–compensation balance, so the land use conversion in Hohhot was relatively stable throughout the decade.

As can be seen in Table 11, the land use type conversion in Taiyuan was relatively stable. The construction land area of Taiyuan increased from 488.52 km^2^ to 741.89 km^2^ over ten years, with a growth rate of 51.86%. The increase in construction land was mainly due to occupying cultivated land, and 234.12 km^2^ of cultivated land was converted to construction land over the decade. In addition, 217.47 km^2^ of cultivated land was also converted to grassland, resulting in a 14.72% decline in cultivated land from 2667.84 km^2^ to 2275.21 km^2^.

As can be seen from Table 12, the major land use type conversions in Xi’an between 2010 and 2020 were grassland and water area. Among them, 30.95% of the grassland was converted to forest land, totaling 52.46 km^2^; 26.03% of the water area was converted to cultivated land, totaling 12.47 km^2^. The construction land area of Xi’an increased from 1216.24 km^2^ to 1395.67 km^2^, with a growth rate of 14.75%. The increase in construction land was mainly due to occupying cultivated land, and 306.93 km^2^ of cultivated land was converted to construction land. Since 127.07 km^2^ of construction land was converted into cultivated land, the land use conversion in Xi’an was relatively stable over the decade.

As can be seen from Table 13, the major land use type conversions in Zhengzhou between 2010 and 2020 were grassland and water area: 29.45% of the grassland was converted to forest land, totaling 28.59 km^2^; 58.67% of the water area was converted to cultivated land, totaling 72.64 km^2^. The construction land area of Zhengzhou increased from 1235.84 km^2^ to 2051.59 km^2^, with a growth rate of 66.01%. The increase in construction land was mainly due to occupying cultivated land, and 887.01 km^2^ of cultivated land was converted to construction land. However, the conversions of other land types to arable land were relatively minor, meaning that the area of cultivated land in Zhengzhou showed a declining trend over the decade.

As can be seen in Table 14, the major land use type conversions in Jinan between 2010 and 2020 were water area and bare land: 25.89% of the water area was converted to cultivated land, totaling 39.22 km^2^; 21.07% of the bare land was converted to forest land, totaling 6.86 km^2^. The construction land area increased by 60.97%, from 1358.76 km^2^ to 2187.25 km^2^. The increase in construction land was mainly due to occupying cultivated land, and 956.28 km^2^ of cultivated land was converted to construction land. Although 159.59 km^2^ of construction land was converted to cultivated land, the area of cultivated land occupied by construction land in Jinan was still substantial.

The three cities of Xining, Lanzhou and Yinchuan are located in the upper reaches of the Yellow River Basin, where grassland was the most dominant land use type, followed by cultivated land. Grassland accounted for 52.42% of the total area of the three upstream cities. The changing trends of different land use types in the three upstream cities were as follows: the area of cultivated land and grassland gradually decreased while the area of forest land decreased to a smaller extent; the area of construction land gradually increased, and the area of water area, bare land and glacial snow increased to a smaller extent.

The three cities of Hohhot, Taiyuan and Xi’an are located in the middle reaches of the Yellow River Basin. Cultivated land was the most dominant land use type, which accounted for 41.94% of the total area of the three midstream cities, followed by forest land. The changing trends of different land use types in the three midstream cities were as follows: the area of cultivated land gradually decreased, while the area of grassland decreased to a smaller extent; the area of construction land gradually increased, and the area of forest land increased to a smaller extent; the area of water area increased slightly and the area of bare land decreased slightly.

The two cities of Zhengzhou and Jinan are located in the lower reaches of the Yellow River Basin. Cultivated land was the most dominant land use type, which accounted for 64.44% of the total area of the two downstream cities, followed by construction land. This pattern is consistent with the findings of Zhang et al. (2020) [65] The changing trends of different land use types in the two downstream cities were as follows: the area of cultivated land gradually decreased while the area of water area decreased to a smaller extent; the area of construction land gradually increased, while the area of forest land and grassland increased to a smaller extent; the area of bare land decreased slightly.

The eight central cities in the Yellow River Basin showed geographical variation in land use type transfer. The main reason for this is that the topography of the upstream areas is suitable for the development of grassland, while the topography of the midstream and downstream regions is suitable for cultivation. In addition, due to the increasing land demands stemming from economic development, the area of construction land in the upstream, midstream and downstream areas is continuously growing, thus encroaching on the area of cultivated land and grassland. Although the increase in the area of construction land promotes the urban economy, it also erodes cultivated land to a certain extent, thus affecting regional ecological development and SLU.

From 2010 to 2020, the major significant changes in land use types in the central cities of the Yellow River Basin occurred in construction land and cultivated land, with an increase of 3687.47 km^2^ and a decrease of 3419.73 km^2^, respectively. In terms of the transfer process of land use types in these Yellow River Basin cities, the transfer from cultivated land to other types of land use played a major role, while construction land showed a significant expansion over the past ten years. This pattern is consistent with the findings of Zhang et al. (2020) [65]. With the rapid economic development of the Yellow River Basin in the past ten years, population migration and economic development have been accelerating urbanization, inducing a growing area of construction land. As the land resources are limited, substantial cultivated land has been taken up for construction, leaving a growing imbalance between construction land and cultivated land. As the Yellow River Basin carries the burden of ecological protection and food production alongside economic development, the state should strengthen the SLU in ecologically fragile regions and protect cultivated land. The state should not focus on economic growth only to underestimate the importance of ecological protection and SLU.

## 5. Conclusions and Discussion

Investigating the central cities of the Yellow River Basin, this paper applied the entropy method, weighted method and obstacle factor analysis method to the selection of eighteen indexes in three categories (economic feasibility, social acceptability and resource and environmental sustainability) and the construction of an evaluation index system for SLU. The paper then carried out a comprehensive evaluation and obstacle factor diagnosis study on SLU in the central cities of the Yellow River Basin, and obtained the following basic conclusions:

(1) From 2009 to 2018, the SLU level in the central cities of the Yellow River Basin evolved from the “Unsustainable Level” to the “Initial Sustainable Level” and then to the “Basic Sustainable Level”. The overall development trend was positive, and the level of SLU became higher. During the period 2009–2012, the level of SLU in the central cities of the Yellow River Basin was at the “Unsustainable Level”; the level of SLU in the central cities of the Yellow River Basin was significantly improved after 2013; until the end of 2018, among the eight cities, only Jinan was at the “Fully Sustainable Level” of the SLU level, and the six cities of Xining, Lanzhou, Yinchuan, Taiyuan, Xi’an and Zhengzhou were at the “Basic Sustainable Level”, while Hohhot was still at the “Initial Sustainable Level”.

(2) From 2009 to 2018, there was significant geographical variation in spatial disparities in SLU in the central cities of the Yellow River Basin. As of 2018, the average comprehensive score of SLU showed a pattern of downstream > upstream > midstream. Jinan achieved the “Fully Sustainable Level”. The level of SLU in cities in the upper and lower reaches of the Yellow River improved significantly faster than that in cities in the middle reaches of the Yellow River. The central cities in the upper reaches of the Yellow River not only emphasized the protection of the ecological environment, but also continuously improved the quality of the development in the context of the market economy. This mode greatly improved the SLU in the cities in the upper reaches of the Yellow River. Advantaged in geographical location and abundant in resources, the cities in the lower reaches of the Yellow River experienced rapid economic development and rapid growth in economic feasibility scores. In 2015, the economic viability scores became the strongest driver of comprehensive land use scores among the eight cities. The economic development greatly improved the level of SLU in the lower reaches of the Yellow River.

(3) A diagnosis of the obstacle degree to SLU in central cities in the Yellow River Basin from 2009 to 2018 concluded that the factors that hindered the improvement of SLU in central cities in the Yellow River Basin in 2009 were focused on resource and environmental sustainability, mainly including the sewage treatment rate, park green area per capita and green coverage rate of built-up areas. The obstacle factors in 2018 were mainly focused on social acceptability, mainly including the population density, grain production and natural population growth rate.

(4) From 2010 to 2020, the major significant changes in land use types in the central cities of the Yellow River Basin took place in construction land and cultivated land, with an increase of 3687.47 km^2^ and a decrease of 3419.73 km^2^, respectively. In terms of the transfer process of land use types in these Yellow River Basin cities, the transfer from cultivated land to other types of land use played a major role, while construction land showed a significant expansion over the past ten years.

In general, to achieve SLU in ecologically fragile areas, it is particularly necessary to consider the ecological and environmental carrying capacity, as well as the suitability of territorial space development [66,67,68,69]. It is also important to take urban development planning as the basic principle, take the speed of urban development and future development direction into account, take the creation of urban characteristics as the basic value orientation, construct the dynamic adjustment mechanism of urban growth boundary, and establish a sound urban space control policy system.

In addition to appropriately “empowering” local governments to enhance their decision-making “flexibility”, effectively correcting the “GDP-only” preference in performance appraisal, optimizing the performance appraisal index system of local governments, and constructing an accountability mechanism for “urban space” decision-making, the government should also establish a sound real estate tax management system, optimize local government revenue channels, construct a sustainable revenue realization mechanism for local governments, guide the reasonable return of local governments’ “land management functions”, carry out land improvement, and construct land increase and decrease linkage actions suitable for each region.

It is essential to comprehensively consider the four dimensions of ecology, economy, society and morphology, and to significantly improve the efficiency of urban spatial resource allocation. It is also of vital importance to continue to improve the performance of urban spatial organization and operation and the overall carrying capacity of the city. Policy makers are also expected to reasonably predict the evolution of urban space, strictly hold the “ecological bottom line” of urban space utilization [70], build a real-time urban space ecological monitoring system [71], actively promote the construction of “ecological city” [72,73], and significantly improve the city’s sustainable operation capabilities.

## Figures and Tables

**Table 1:** Comprehensive evaluation index system for sustainable land use (SLU) in central cities of the Yellow River Basin, China.

Target Layer	Rule Layer	Index Layer	Unit	Index Properties
Sustainable land use	Social acceptability	Natural population growth rateX_1_	‰	−
		Population densityX_2_	Person/km^2^	−
		Urbanization rate of resident populationX_3_	%	+
		Grain productionX_4_	10 thousand tons	+
		Disposable income per capitaX_5_	Yuan/person	+
		Engel coefficientX_6_	%	−
	Economic feasibility	GDP per capitaX_7_	Yuan/person	+
		Investment in fixed assetsX_8_	in CNY 100 million	+
		Total retail sales of consumer goodsX_9_	in CNY 100 million	+
		Public finance budget revenueX_10_	CNY 10 thousand	+
		Share of tertiary sector in GDPX_11_	%	+
		Real estate development investmentX_12_	in CNY 100 million	+
	Resource and environmental sustainability	Green coverage rate of built-up areasX_13_	%	+
		Sewage treatment rateX_14_	%	+
		Industrial solid waste generationX_15_	10 thousand tons	−
		Park green area per capitaX_16_	m^2^/person	+
		Fertilizer application amountX_17_	tons	−
		Water resources per capitaX_18_	m^3^/person	+

**Table 2:** Evaluation index weights for SLU in central cities of the Yellow River Basin, China.

Index	Xining	Lanzhou	Yinchuan	Hohhot	Taiyuan	Xi’an	Zhengzhou	Jinan
X_1_	0.040	0.037	0.039	0.062	0.084	0.044	0.037	0.033
X_2_	0.041	0.064	0.091	0.042	0.090	0.024	0.048	0.039
X_3_	0.034	0.036	0.035	0.037	0.038	0.055	0.055	0.193
X_4_	0.048	0.045	0.047	0.061	0.036	0.077	0.026	0.047
X_5_	0.048	0.049	0.059	0.043	0.049	0.038	0.044	0.047
X_6_	0.078	0.062	0.046	0.062	0.085	0.115	0.060	0.069
X_7_	0.039	0.041	0.052	0.038	0.068	0.048	0.045	0.044
X_8_	0.059	0.035	0.053	0.049	0.064	0.054	0.052	0.064
X_9_	0.055	0.044	0.048	0.043	0.050	0.054	0.048	0.054
X_10_	0.048	0.048	0.042	0.094	0.048	0.047	0.041	0.048
X_11_	0.065	0.071	0.100	0.066	0.081	0.093	0.073	0.049
X_12_	0.051	0.048	0.043	0.065	0.042	0.055	0.055	0.048
X_13_	0.062	0.091	0.048	0.081	0.041	0.040	0.055	0.038
X_14_	0.042	0.057	0.027	0.028	0.024	0.035	0.069	0.026
X_15_	0.075	0.080	0.061	0.054	0.025	0.060	0.060	0.020
X_16_	0.043	0.057	0.058	0.072	0.042	0.031	0.122	0.054
X_17_	0.130	0.069	0.059	0.057	0.091	0.042	0.053	0.086
X_18_	0.041	0.066	0.093	0.047	0.042	0.089	0.057	0.042

**Table 3:** Evaluation criteria for SLU level.

Score	Evaluation Criteria
0 ≤ F < 0.5	Unsustainable Level
0.5 < F < 0.7	Initial Sustainable Level
0.7 ≤ F ≤ 0.85	Basic Sustainable Level
0.85 < F ≤ 1	Fully Sustainable Level

**Table 4:** Annual comprehensive score of SLU indexes for central cities in the Yellow River Basin, China.

City	2009	2010	2011	2012	2013	2014	2015	2016	2017	2018
Xining	0.288	0.236	0.304	0.359	0.367	0.434	0.519	0.737	0.807	0.840
Lanzhou	0.193	0.177	0.234	0.353	0.463	0.414	0.452	0.609	0.710	0.803
Yinchuan	0.455	0.348	0.314	0.403	0.364	0.406	0.483	0.587	0.576	0.707
Hohhot	0.213	0.256	0.286	0.439	0.542	0.555	0.533	0.690	0.593	0.534
Taiyuan	0.213	0.203	0.218	0.285	0.362	0.488	0.586	0.637	0.707	0.726
Xi’an	0.274	0.311	0.398	0.363	0.418	0.443	0.525	0.649	0.676	0.725
Zhengzhou	0.299	0.292	0.294	0.240	0.297	0.366	0.442	0.543	0.708	0.803
Jinan	0.185	0.260	0.263	0.319	0.362	0.360	0.484	0.673	0.709	0.865

**Table 5:** Score of each rule layer for SLU in central cities of the Yellow River Basin, China.

City	Rule Layer	2009	2010	2011	2012	2013	2014	2015	2016	2017	2018
Xining	Social acceptability	0.115	0.099	0.122	0.149	0.136	0.107	0.147	0.192	0.188	0.178
	Economic feasibility	0.047	0.062	0.076	0.104	0.127	0.171	0.215	0.262	0.281	0.306
	Resource and Environmental Sustainability	0.126	0.075	0.106	0.106	0.104	0.156	0.157	0.283	0.337	0.356
Lanzhou	Social acceptability	0.119	0.106	0.166	0.185	0.150	0.146	0.158	0.134	0.150	0.167
	Economic feasibility	0.005	0.016	0.039	0.072	0.121	0.160	0.197	0.235	0.242	0.274
	Resource and Environmental Sustainability	0.069	0.055	0.029	0.096	0.192	0.109	0.096	0.240	0.319	0.362
Yinchuan	Social acceptability	0.178	0.111	0.124	0.121	0.127	0.118	0.141	0.124	0.129	0.180
	Economic feasibility	0.048	0.059	0.068	0.101	0.143	0.174	0.215	0.259	0.277	0.299
	Resource and Environmental Sustainability	0.230	0.178	0.122	0.181	0.094	0.113	0.127	0.204	0.170	0.228
Hohhot	Social acceptability	0.069	0.083	0.060	0.141	0.147	0.162	0.182	0.177	0.224	0.230
	Economic feasibility	0.004	0.051	0.111	0.195	0.275	0.218	0.243	0.280	0.188	0.151
	Resource and Environmental Sustainability	0.141	0.123	0.115	0.102	0.119	0.176	0.107	0.233	0.181	0.153
Taiyuan	Social acceptability	0.135	0.105	0.094	0.097	0.110	0.165	0.196	0.180	0.274	0.191
	Economic feasibility	0.012	0.030	0.055	0.104	0.153	0.204	0.268	0.300	0.241	0.294
	Resource and Environmental Sustainability	0.066	0.068	0.070	0.084	0.098	0.118	0.123	0.158	0.192	0.241
Xi’an	Social acceptability	0.148	0.172	0.177	0.172	0.158	0.136	0.138	0.182	0.170	0.223
	Economic feasibility	0.001	0.027	0.061	0.102	0.144	0.184	0.234	0.267	0.319	0.350
	Resource and Environmental Sustainability	0.125	0.112	0.160	0.089	0.116	0.124	0.153	0.199	0.188	0.152
Zhengzhou	Social acceptability	0.110	0.097	0.093	0.108	0.120	0.124	0.148	0.133	0.131	0.166
	Economic feasibility	0.007	0.021	0.042	0.073	0.107	0.137	0.174	0.221	0.263	0.312
	Resource and Environmental Sustainability	0.182	0.173	0.160	0.059	0.070	0.104	0.121	0.189	0.314	0.325
Jinan	Social acceptability	0.113	0.124	0.129	0.134	0.107	0.095	0.152	0.272	0.260	0.323
	Economic feasibility	0.000	0.035	0.055	0.089	0.125	0.157	0.192	0.230	0.268	0.306
	Resource and Environmental Sustainability	0.072	0.101	0.079	0.096	0.129	0.107	0.140	0.171	0.181	0.236

**Table 6:** Obstacle degree to SLU Indexes in Central Cities of the Yellow River Basin, China.

Year	City	Xining	Lanzhou	Yinchuan	Hohhot	Taiyuan	Xi’an	Zhengzhou	Jinan
2009	Index	X_14_	X_9_	X_14_	X_14_	X_14_	X_16_	X_13_	X_14_
	OD (%)	30.7	21.39	32.2	25.8	29.02	29.92	23.08	35.74
2010	Index	X_14_	X_14_	X_10_	X_11_	X_3_	X_3_	X_13_	X_13_
	OD (%)	29.91	20.85	23.95	22.92	23.28	22.75	21.61	20.63
2011	Index	X_13_	X_14_	X_16_	X_11_	X_15_	X_15_	X_13_	X_14_
	OD (%)	18.33	21.6	23.21	22.96	34.41	18.46	20.88	19.53
2012	Index	X_13_	X_11_	X_16_	X_6_	X_6_	X_17_	X_14_	X_6_
	OD (%)	15.96	17.51	17.46	21.46	19.53	16.66	21.3	15.27
2013	Index	X_18_	X_3_	X_18_	X_1_	X_6_	X_11_	X_14_	X_6_
	OD (%)	20.84	32.67	17.55	15.87	17.67	14.32	20.37	14.85
2014	Index	X_1_	X_3_	X_13_	X_10_	X_1_	X_17_	X_14_	X_1_
	OD (%)	20.7	33.44	21.49	13.99	14.08	22.29	20.37	26.63
2015	Index	X_1_	X_2_	X_13_	X_18_	X_4_	X_17_	X_14_	X_18_
	OD (%)	17.25	17.82	17.65	16.3	17.87	18	19.44	19.26
2016	Index	X_18_	X_2_	X_1_	X_2_	X_1_	X_1_	X_15_	X_1_
	OD (%)	16.54	18.32	20.24	15.38	13.84	20.34	18.44	17.95
2017	Index	X_2_	X_4_	X_1_	X_18_	X_8_	X_1_	X_4_	X_18_
	OD (%)	17.86	19.97	23.53	18.17	16.15	31.45	30.04	23.29
2018	Index	X_2_	X_4_	X_15_	X_2_	X_4_	X_2_	X_1_	X_15_
	OD (%)	22.32	20.91	16.79	21.15	21.03	38.11	22.11	53.7

Note: OD (%) stands for Obstacle Degree (%).

**Table 7:** The land use transfer matrix in Xining from 2010 to 2020 (unit: km^2^).

	2010	Cultivated Land	Forest Land	Grassland	Water Area	Construction Land	Bare Land	Glacial Snow	Total
2020									
Cultivated land		2473.46	1.29	92.06	1.87	7.75	0.01	0	2576.44
Forest land		6.37	3.83	45.23	0.08	0.04	0	0	55.54
Grassland		104.03	2.74	4119.14	1.57	1.13	8.28	17.18	4254.07
Water area		3.02	0.23	1.52	9.78	0.33	0	0	14.88
Construction land		257.10	0.32	39.49	1.50	159.23	0.09	0	457.73
Bare land		0.01	0	56.77	0	0	51.23	4.82	112.83
Glacial snow		0	0	107.25	0	0	0.94	47.11	155.30
Total		2843.99	8.41	4461.46	14.79	168.48	60.55	69.10	7626.79

**Table 8:** The land use transfer matrix in Lanzhou from 2010 to 2020 (unit: km^2^).

	2010	Cultivated Land	Forest Land	Grassland	Water Area	Construction Land	Bare Land	Total
2020								
Cultivated land		4532.75	11.77	377.16	4.73	6.15	5.05	4937.61
Forest land		23.35	61.03	87.50	0.02	0	0	171.89
Grassland		360.12	156.76	6584.34	0.52	1.11	29.69	7132.54
Water area		8.82	0.51	2.16	28.15	0.58	0.02	40.22
Construction land		450.69	0.60	122.33	2.09	191.95	2.21	769.87
Bare land		5.89	0.01	21.13	0	0.02	33.41	60.46
Total		5381.62	230.67	7194.61	35.51	199.80	70.38	13,112.58

**Table 9:** The land use transfer matrix in Yinchuan from 2010 to 2020 (unit: km^2^).

	2010	Cultivated Land	Forest Land	Grassland	Water Area	Construction Land	Bare Land	Total
2020								
Cultivated land		2428.14	3.44	120.59	36.89	31.51	12.43	2632.99
Forest land		0.72	116.24	55.62	0.13	0.09	0.56	173.36
Grassland		52.14	54.72	3096.53	4.13	2.54	63.41	3273.47
Water area		54.28	2.68	16.01	70.20	0.34	0.54	144.05
Construction land		295.74	8.10	137.32	3.27	198.28	24.61	667.31
Bare land		3.07	4.86	97.35	1.42	0.96	226.44	334.11
Total		2834.09	190.05	3523.41	116.03	233.72	327.98	7225.28

**Table 10:** The land use transfer matrix in Hohhot from 2010 to 2020 (unit: km^2^).

	2010	Cultivated Land	Forest Land	Grassland	Water Area	Construction Land	Bare Land	Total
2020								
Cultivated land		7391.85	47.54	809.69	24.33	129.57	5.30	8408.28
Forest land		15.03	1273.91	206.16	0.39	1.67	1.07	1498.24
Grassland		786.64	213.55	4742.92	16.28	17.29	15.64	5792.32
Water area		23.33	0.62	7.16	96.14	0.76	0.10	128.11
Construction land		352.12	7.13	105.10	1.71	572.93	0.82	1039.82
Bare land		4.73	1.18	12.04	0.06	0.07	7.88	25.96
Total		8573.70	1543.94	5883.07	138.90	722.30	30.82	16,892.73

**Table 11:** The land use transfer matrix in Taiyuan from 2010 to 2020 (unit: km^2^).

	2010	Cultivated Land	Forest Land	Grassland	Water Area	Construction Land	Total
2020							
Cultivated land		2080.49	40.55	135.11	2.14	16.92	2275.21
Forest land		217.47	1649.01	275.92	0.50	0.59	2143.49
Grassland		130.81	256.66	1450.73	1.08	2.18	1841.46
Water area		4.95	1.18	2.38	38.61	0.95	48.08
Construction land		234.12	5.84	33.09	0.94	467.89	741.89
Total		2667.84	1953.24	1897.24	43.28	488.52	7050.11

**Table 12:** The land use transfer matrix in Xi’an from 2010 to 2020 (unit: km^2^).

	2010	Cultivated Land	Forest Land	Grassland	Water Area	Construction Land	Total
2020							
Cultivated land		3427.40	43.22	16.16	12.47	127.07	3626.31
Forest land		40.03	4816.84	52.46	1.15	0.59	4911.07
Grassland		17.09	73.22	97.11	0.70	4.13	192.25
Water area		18.58	1.86	0.99	33.02	0.52	54.97
Construction land		306.93	1.59	2.64	0.57	1083.94	1395.67
Bare land		0	0.03	0.13	0	0	0.16
Total		3810.03	4936.76	169.49	47.92	1216.24	10,180.44

**Table 13:** The land use transfer matrix in Zhengzhou from 2010 to 2020 (unit: km^2^).

	2010	Cultivated Land	Forest Land	Grassland	Water Area	Construction Land	Total
2020							
Cultivated land		4209.84	29.69	6.63	72.64	90.85	4409.65
Forest land		29.49	580.14	28.59	0.03	0.93	639.19
Grassland		14.36	28.18	58.96	1.66	0.70	103.86
Water area		20.81	0.22	0.12	36.47	1.18	58.80
Construction land		887.01	6.59	2.79	13.01	1142.18	2051.59
Total		5161.51	644.83	97.09	123.81	1235.84	7263.08

**Table 14:** The land use transfer matrix in Jinan from 2010 to 2020 (unit: km^2^).

	2010	Cultivated Land	Forest Land	Grassland	Water Area	Construction Land	Bare Land	Total
2020								
Cultivated land		7435.70	81.67	80.87	39.22	159.59	2.10	7799.15
Forest land		164.42	578.85	79.23	0.45	4.29	6.86	834.10
Grassland		183.69	93.67	360.31	1.31	3.54	4.25	646.77
Water area		70.48	0.72	4.12	107.32	2.91	1.82	187.37
Construction land		956.28	12.16	26.46	3.10	1188.43	0.82	2187.25
Bare land		2.04	6.34	2.97	0.07	0.01	16.69	28.11
Total		8812.59	773.41	553.96	151.47	1358.76	32.55	11,682.75

## Data Availability

The data presented in this study for the eight central cities in the study period were obtained from the Statistical Yearbooks, the Water Resources Bulletin and the Statistical Bulletin of National Economic and Social Development. ArcGIS remote sensing images, the vector boundary data of eight cities and the Chinese altitude (DEM) spatial distribution data were obtained from https://www.resdc.cn, (accessed on 3 March 2022).
